# Cost-Effectiveness Analysis of Sex-Stratified *Plasmodium vivax* Treatment Strategies Using Available G6PD Diagnostics to Accelerate Access to Radical Cure

**DOI:** 10.4269/ajtmh.19-0943

**Published:** 2020-05-04

**Authors:** Angela Devine, Rosalind E. Howes, David J. Price, Kerryn A. Moore, Benedikt Ley, Julie A. Simpson, Sabine Dittrich, Ric N. Price

**Affiliations:** 1 Division of Global and Tropical Health, Menzies School of Health Research, Charles Darwin University, Darwin, Australia;; 2 Centre for Epidemiology and Biostatistics, Melbourne School of Population and Global Health, The University of Melbourne, Melbourne, Australia;; 3 Malaria and Fever Programme, Foundation for Innovative New Diagnostics (FIND), Geneva, Switzerland;; 4 Big Data Institute, Nuffield Department of Medicine, University of Oxford, Oxford, United Kingdom;; 5 Victorian Infectious Diseases Reference Laboratory Epidemiology Unit at the Peter Doherty Institute for Infection and Immunity, The University of Melbourne and Royal Melbourne Hospital, Melbourne, Australia;; 6 Department of Infectious Disease Epidemiology, London School of Hygiene and Tropical Medicine, London, United Kingdom;; 7 Infection and Immunity, Murdoch Children’s Research Institute, Melbourne, Australia;; 8 Centre for Tropical Medicine and Global Health, Nuffield Department of Clinical Medicine, University of Oxford, Oxford, United Kingdom;; 9 Mahidol-Oxford Tropical Medicine Research Unit, Faculty of Tropical Medicine, Mahidol University, Bangkok, Thailand

## Abstract

Tafenoquine has been licensed for the single-dose radical cure of *Plasmodium vivax* in adults; however, it is only recommended in patients with > 70% of normal glucose-6-phosphate dehydrogenase (G6PD) activity. Because this may hinder widespread use, we investigated sex-based treatment strategies in which all adult patients are tested with a qualitative G6PD rapid diagnostic test (RDT). Glucose-6-phosphate dehydrogenase normal males are prescribed tafenoquine in all three strategies, whereas G6PD normal females are prescribed either a low-dose 14-day primaquine regimen (PQ14, total dose 3.5 mg/kg) or a high-dose 7-day primaquine regimen (PQ7, total dose 7 mg/kg), or referred to a healthcare facility for quantitative G6PD testing before prescribing tafenoquine. Patients testing G6PD deficient are prescribed a weekly course of primaquine for 8 weeks. We compared the cost-effectiveness of these three strategies to usual care in four countries using a decision tree model. Usual care in Ethiopia does not include radical cure, whereas Afghanistan, Indonesia, and Vietnam prescribe PQ14 without G6PD screening. The cost per disability-adjusted life-year (DALY) averted was expressed through incremental cost-effectiveness ratios (ICERs). Compared with usual care, the ICERs for a sex-based treatment strategy with PQ7 for females from a healthcare provider perspective were $127 per DALY averted in Vietnam, $466 in Ethiopia, $1,089 in Afghanistan, and $4,443 in Indonesia. The PQ14 and referral options cost more while averting fewer DALYs than PQ7. This study provides an alternative cost-effective mode of rolling out tafenoquine in areas where initial testing with only a G6PD RDT is feasible.

## INTRODUCTION

Outside sub-Saharan Africa, *Plasmodium vivax* is a common cause of human malaria, with an estimated 14.3 million cases in 2017.^1^ The only available drugs that kill the dormant liver stages (hypnozoites) of *P. vivax* are the 8-aminoquinoline compounds primaquine and tafenoquine. The current WHO treatment guidelines recommend that primaquine is administered as a 14-day regimen at a total dose of 3.5 mg/kg (PQ14); however, a higher dose (total dose 7 mg/kg) is recommended in areas with a high risk of recurrence.^2^ The prolonged treatment course extends long after symptomatic recovery and in practice is associated with poor patient adherence and low effectiveness.^3,4^ Clinical trials have explored ways of shortening the course of primaquine without compromising safety or efficacy. Two recent studies have shown that a 7-day high-dose primaquine regimen (PQ7, total dose 7 mg/kg) was well tolerated with a similar efficacy to the same total dose administered more than 14 days.^5,6^ Although a high-dose short-course 7-day regimen may improve adherence, it has yet to be implemented into policy. For the purpose of this study, PQ14 corresponds to the low-dose regimen and PQ7 to the high-dose regimen.

In 2018, tafenoquine became the first new drug to be licensed for the radical cure of *vivax* malaria in over 60 years. It has the major advantage of being administered as a single-dose treatment. Clinical trials have shown that when combined with chloroquine, tafenoquine is non-inferior to the low-dose primaquine regimen (3.5 mg/kg total dose).^7,8^ Both drugs can cause severe hemolysis in individuals with glucose-6-phosphate dehydrogenase (G6PD) deficiency.

Glucose-6-phosphate dehydrogenase deficiency is an inherited X-linked enzymopathy present in up to 20% of malaria-endemic populations.^9^ Males have a single copy of the gene and are either hemizygous G6PD deficient with < 30% of normal G6PD activity or normal.^10^ By contrast, females have two copies of the gene and can be homozygous G6PD normal or deficient, or heterozygous. Glucose-6-phosphate dehydrogenase activity is measured in U/gHb; however, to date, no universal cutoff exists to guide primaquine and tafenoquine treatment. Instead, a population-specific median is calculated and defined as “100% activity.”^11^ Most, if not all, qualitative G6PD diagnostics are designed to discriminate between individuals greater than or less than the 30% threshold, a dichotomy not suitable to identify heterozygous females.^11,12^ In heterozygous females, random inactivation of one of the *G6PD* genes, through a process known as lyonization, results in a mixture of G6PD normal and deficient red blood cells with a combined intermediate phenotype typically ranging between 30% and 70% G6PD activity; these individuals are at the risk of hemolysis.^13^ Tafenoquine is slowly eliminated with a half-life of approximately 14 days: it cannot be stopped in the event of a hemolytic reaction and so to avoid hemolysis in heterozygous females, it is only recommended in patients with greater than 70% G6PD activity. Because primaquine is rapidly eliminated from the body with a half-life of 6 hours, the criteria for its use are less stringent, and most eligibility guidelines set the threshold for defining G6PD deficiency at 30%.^10^

To reduce the risk of drug-induced hemolysis, patients should be tested for G6PD deficiency before prescribing an 8-aminoquinoline drug.^14^ This can be achieved with either qualitative or quantitative tests.^12^ Qualitative G6PD diagnostics categorize individuals as normal or deficient; however, these tests are only reliable at a threshold of about 30% enzyme activity and thus label heterozygous females with > 30% G6PD activity as normal. Quantitative tests provide a continuous measure of G6PD activity and can therefore identify individuals with activity less than 70%.^15^ Several point-of-care tests for G6PD deficiency have been developed. The qualitative rapid diagnostic test (RDT), CareStart™ G6PD test (Access Bio Inc., Somerset, NJ), has 96% sensitivity at the 30% threshold, with a negative predictive value of 99%.^16^ The only available point-of-care quantitative tests are the STANDARD™ G6PD (SD Biosensor, Suwon, South Korea) and CareStart Biosensor (AccessBio). These tests include a handheld analyzer device and test strips while requiring additional training to prepare samples, and interpret and use test results. Although tafenoquine will overcome the limitations of poor adherence, the labeling necessitates the use of a quantitative G6PD test, which is likely to restrict its widespread use while incurring the additional costs associated with quantitative G6PD testing.

Radical cure reduces recurrent illness in patients, and, if used widely, has potential to impact on ongoing transmission with community benefits.^17^ Previous research has indicated that strategies using high-dose 14-day primaquine after screening with a G6PD RDT would be cost-effective on the Thai–Myanmar border, but results were dependent on primaquine adherence. In this current study, we investigated the cost-effectiveness of a novel approach of deploying tafenoquine through a sex-based treatment strategy that maximizes the proportion of patients receiving shortened doses of radical cure. In this strategy, tafenoquine is prescribed to males who screen G6PD normal by the qualitative RDT. Females who test G6PD normal could be prescribed either PQ7 or PQ14, or be referred to a higher healthcare facility for quantitative testing to assess eligibility for tafenoquine treatment. To investigate the potential economic impact of this sex-based treatment strategy, we quantified the costs and effectiveness of each option of the treatment strategy compared with usual care in four countries with endemic *P. vivax*.

## MATERIALS AND METHODS

### Model structure and probabilities.

An economic evaluation was undertaken adapting a previously published decision tree model of *P. vivax* management^18^ to compare the cost-effectiveness of treatment strategies for radical cure (routine care and sex-based treatment) in terms of cost per disability-adjusted life-year (DALY) averted. All analyses were conducted in R version 3.6.1, and the model was parameterized for Afghanistan, Ethiopia, Indonesia, and Vietnam using data from a recent clinical trial^5^ and literature review. A time horizon of 1 year was chosen to reflect the duration of follow-up in the trial and because previous analyses have shown that the risk of recurrent *P. vivax* beyond 12 months falls to a level similar to that due to reinfection alone.^4^ All model parameters have a base case value with a range used for the sensitivity analyses, which was taken from CIs or plausible estimated values when data were not available (Tables 1 and 2).

**Table 1:** Country-specific model parameters (all costs are in 2016 US)

Parameter	Afghanistan		Ethiopia		Indonesia		Vietnam		Distribution	Reference
	Base	Range	Base	Range	Base	Range	Base	Range		
Proportion of adult patients who are male	0.76	0.5–1.0	0.63	0.5–1.0	0.59	0.5–1.0	0.88	0.5–1.0	Beta	5
Proportion who have at least 1 vivax malaria recurrence if not treated with radical cure	0.43	0.33–0.54	0.56	0.47–0.65	0.34	0.28–0.41	0.55	0.42–0.67	Beta	5
Relative risk of having at least 1 recurrence if prescribed PQ7	0.35	0.25–0.50	0.24	0.18–0.32	0.29	0.22–0.39	0.18	0.12–0.28	Lognormal	5
Number of recurrences over 1 year without radical cure (if have at least 1 recurrence)	1.56	1.43–1.69	2.05	1.89–2.21	1.57	1.47–1.67	1.94	1.74–2.14	Normal	5
Number of recurrences over 1 year with PQ7 (if have at least 1 recurrence)	1.23	1.17–1.29	1.21	1.15–1.27	1.10	1.07–1.13	1.23	1.15–1.31	Normal	5
Proportion of males with G6PD deficiency (< 30% activity)	0.07	0.06–0.10	0.01	0.01–0.02	0.07	0.05–0.09	0.05	0.02–0.11	Beta	9
Proportion of females with G6PD deficiency (< 30% activity)	0.04	0.03–0.06	0.01	0.00–0.01	0.04	0.03–0.06	0.03	0.01–0.06	Exponential	9
Cost of G6PD screening by RDT	$3.42	$1.71–$5.13	$3.56	$1.78–$5.34	$15.34	$7.67–$23.01	$1.67	$0.84–$2.51	Gamma	±50%^26^
Cost of quantitative G6PD screening	$6.62	$3.31–$9.93	$7.18	$3.59–$10.77	$12.26	$6.13–$18.40	$3.18	$1.59–$4.77	Gamma	Assumptions with ±50%^26,27^
Cost of PQ14	$0.19	$0.10–$0.29	$0.43	$0.22–$0.65	$0.43	$0.22–$0.65	$0.43	$0.22–$0.65	Gamma	±50%^26^
Cost per malaria episode	$3.43	$1.72–$5.15	$5.28	$2.64–$7.92	$6.18	$3.09–$9.27	$5.58	$2.79–$8.37	Gamma	±50%^26^
Cost per severe malaria episode	$29.2	$14.6–$43.8	$17.6	$8.8–$26.4	$155.8	$77.9–$233.7	$73.8	$36.9–$110.7	Gamma	±50%^27^
Household cost per *vivax* episode	$8.2	n/a	$11.1	n/a	$50.8	n/a	$23.6	n/a	Gamma	Scenario analysis only^26^
Household travel cost per referral	$2.9	n/a	$1.8	n/a	$1.4	n/a	$2.8	n/a	Gamma	Scenario analysis only^26^
Cost per hemolytic event	$52.0	$26.0–$78.0	$39.4	$19.7–$59.1	$180.9	$90.5–$271.4	$98.9	$49.5–$148.4	Gamma	±50% for 7-day inpatient stay at a primary hospital and one unit of blood^27,28^
Life expectancy for males, years	43.5	34.8–52.2	49.7	39.8–59.6	40.7	32.6–48.8	49.3	39.4–59.2	Gamma	±20%^30^
Life expectancy for females, years	46.2	37.0–55.4	52.5	42.0–63.0	44.3	35.4–53.2	53	42.4–63.6	Gamma	±20%^30^

G6PD = glucose-6-phosphate dehydrogenase; PQ7 = 7 mg/kg primaquine over 7 days; RDT = rapid diagnostic test.

**Table 2:** Model parameters used in all countries

Parameter	Base	Range	Distribution	Reference
Adherence to PQ7 regimen	0.62	0.25–0.95	Beta	23–25
Adherence to PQ14 regimen	0.47	0.19–0.71	Beta	Assumption that PQ7 adherence would be reduced by 25% for PQ14
Adherence to PQ8W regimen	0.31	0.13–0.48	Beta	Assumption that PQ7 adherence would be reduced by 50% for PQ8W
Proportion of women who uptake referral to a higher facility for quantitative G6PD testing	0.5	0.25–1.00	Beta	Assumption
Relative increase in recurrences if PQ14 or TQ (instead of PQ7) was taken	2.34	1.76–2.93	Beta	±25%^5,22^
RDT sensitivity in males	0.98	0.90–1.00	Beta	30% cutoff^16^
RDT specificity in males	0.97	0.90–1.00	Beta	30% cutoff^16^
RDT sensitivity in females	0.90	0.80–0.96	Beta	30% cutoff^16^
RDT specificity in females	0.68	0.50–0.97	Beta	30% cutoff^16^
Quantitative test sensitivity	0.95	0.89–0.98	Beta	70% cutoff^20^
Quantitative test specificity	0.82	0.68–0.91	Beta	70% cutoff^20^
Proportion of G6PD patients who need a transfusion because of hemolysis after taking radical cure	0.109	0.007–0.15	Beta	Includes PQ7, PQ14, and tafenoquine^40^
Proportion who need a transfusion because of hemolysis but do not receive it	0.1	0.01–0.15	Beta	18
Risk of death due to not receiving a transfusion	0.1	0.01–0.5	Beta	18
Proportion of recurrences that are severe	0.02	0.013–0.027	Beta	41
Proportion of recurrences that result in death	0.0001	0–0.001	Beta	18
Cost of TQ	$2	$1.4-$3.0	Gamma	Assumption
Length of illness: uncomplicated malaria	3 days	1–7 days	Beta	18
Length of illness: severe malaria	7 days	3–10 days	Beta	18
Length of illness: anemia due to malaria	1 month	0.5–2 months	Beta	18
Length of illness: anemia due to severe malaria or hemolysis	3 months	1–6 months	Beta	18
DALY weight for infectious disease: acute episode and moderate	0.053	0.033–0.081	Gamma	29
DALY weight for infectious disease: acute episode and severe	0.210	0.139–0.298	Gamma	29
DALY weight for moderate anemia	0.058	0.038–0.086	Gamma	29
DALY weight for severe anemia	0.164	0.112–0.228	Gamma	29

DALY = disability-adjusted life-year; G6PD = glucose-6-phosphate dehydrogenase; PQ14 = 3.5 mg/kg total primaquine dose over 14 days; PQ7 = 7 mg/kg total primaquine dose over 7 days; PQ8W = 6 mg/kg total primaquine dose weekly for 8 weeks; TQ = tafenoquine; RDT = rapid diagnostic test.

Tafenoquine is currently approved only for patients with vivax malaria aged 16 years and older, and, thus, the economic analysis was undertaken in adults only. The model has different pathways for males and females to reflect differences in G6PD deficiency prevalence, life expectancy, and the diagnostic accuracy of the G6PD tests. The results are presented for the overall adult population excluding pregnant women, who are ineligible for radical cure because of the unknown G6PD status of their fetus.^14^ Breastfeeding women are included in the analysis.^19^ In some locations, the risk of *P. vivax* infection differs between males and females; accordingly, the proportion of males in the model population was derived from the proportion in adults enrolled into the clinical trial.^5^

**Figure 1.**
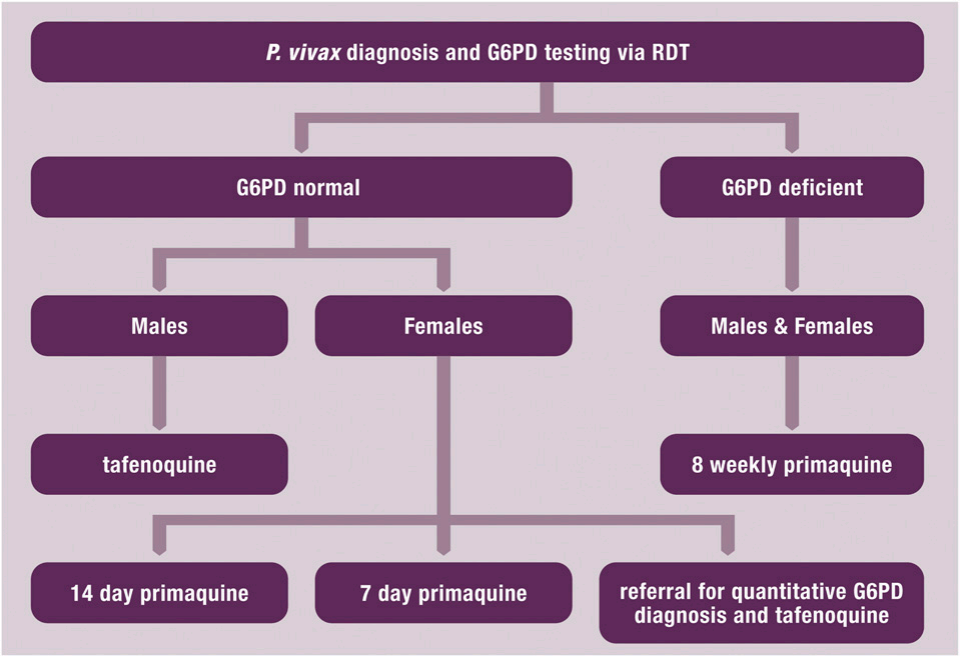
Patient flow diagram for sex-based treatment strategies in a point-of-care setting, with three alternative treatment options for females testing glucose-6-phosphate dehydrogenase (G6PD) normal by rapid diagnostic test (RDT). This figure appears in color at www.ajtmh.org.

Usual care for *P. vivax* malaria is chloroquine plus PQ14 in Afghanistan and Vietnam, DHA-piperaquine plus PQ14 in Indonesia, and chloroquine alone in Ethiopia. Glucose-6-phosphate dehydrogenase testing is not part of routine care in any of these countries. For the sex-based treatment strategy, males with a normal G6PD RDT result would be prescribed tafenoquine, whereas healthcare facilities would choose one of the following three options for the treatment of all females who test G6PD normal by RDT (Figure 1):

1. low-dose PQ14 (3.5 mg/kg total),
2. high-dose PQ7 (7.0 mg/kg total), and
3. referral to a health facility providing quantitative G6PD testing before the prescription of tafenoquine if they have enzyme activity greater than 70%.

In the absence of empirical data, it was assumed that 50% of women would be willing to travel to another facility for further management and that the local clinic would not monitor whether women took up the referral: the impact of this assumption was explored in the sensitivity analysis. Consequently, women who chose not to use their referral would not receive radical cure. The prevalence of G6PD deficiency in males and females was taken from national modeled estimates for the general population.^9^ The diagnostic accuracy and costs of the quantitative G6PD test used for this analysis were taken from the SD Biosensor, given that recent evaluations found this to be the best performing assay.^20^ Individuals testing G6PD deficient at any facility would be given the current WHO-recommended regimen of 8-week course of weekly primaquine (PQ8W, 6 mg/kg total).^14^

### Efficacy and adherence.

The anti-relapse efficacy of tafenoquine was similar to that of PQ14 in South America and the Horn of Africa, but in Southeast Asia, it was lower with tafenoquine than with PQ14.^7^ The clinical efficacy of tafenoquine has yet to be compared with a high-dose primaquine regimen.^21^ It was assumed that the efficacy of tafenoquine was the same when partnered with DHA-piperaquine and chloroquine, although this is yet to be confirmed by phase three clinical trials. The country-specific efficacy of the PQ7 regimen was taken from a large multicentered randomized clinical trial of high-dose primaquine regimens conducted between 2013 and 2018 (IMPROV trial).^5^ In Ethiopia, a separate clinical trial was conducted in a neighboring area (Oromia Region) between 2012 and 2014, which quantified the efficacy of low-dose primaquine.^22^ In this study, patients with low-dose primaquine had 2.34 more recurrences during a year of follow-up than those treated with the high-dose primaquine in Arba Minch and Metahara regions of Ethiopia in the IMPROV trial. This relative difference in the rate of recurrences between low-dose PQ and high-dose PQ was assumed to be the same in Indonesia, Afghanistan, and Vietnam. The PQ8W regimen was assumed to have the same efficacy as PQ14.

The efficacy data were adjusted by adherence rates to calculate the effectiveness of each regimen, although robust estimates of adherence are scarce. A value of 62% was used for the base case analysis of PQ7 based on a study conducted in Peru,^23^ with a range of 25–95% around this estimate taken from the results of two studies of adherence in Thailand.^24,25^ An assumption was made that the adherence to a complete course of treatment was reduced by 25% for PQ14 as compared with PQ7, and PQ8W was reduced by 50% (Table 2). The equations used to calculate the expected number of recurrences are presented in the Supplemental Appendix.

To estimate the proportion of patients who would incorrectly be given radical cure, the prevalence of G6PD deficiency was multiplied by the false-negative rate of the G6PD RDT. Individuals with G6PD deficiency who were treated with weekly primaquine were assumed to not complete a full course of treatment and were assigned the same number of *P. vivax* recurrences as patients who were not prescribed radical cure. Any G6PD-deficient patients erroneously treated with tafenoquine were assumed to have the same number of recurrences as someone who was G6PD normal and treated with tafenoquine.

### Costs, DALYs, and incremental cost-effectiveness ratios (ICERs).

The analysis was conducted from the healthcare provider perspective and reported in 2016 US$. Costs specific to *vivax* malaria were taken from the IMPROV study,^26^ with the exception of the visit costs to the healthcare provider^27^ and blood units required for hemolytic events.^28^ These were varied by 50% in the sensitivity analyses. The cost of G6PD testing included an additional blood sample and a G6PD RDT (Table 1). The cost of a quantitative G6PD test applied in all countries was $4.51, calculated from the following assumptions: a machine cost of $350 with a lifetime of 3 years and a discount rate of 3% in a facility diagnosing 200 patients per year, a test strip cost of $3.50, and quality control costs of $0.39 per patient. Quality control for each batch of 25 test strips included two controls ($1.0 each) and two test strips ($3.50 each) divided by the remaining 23 tests. The PQ7 and PQ8W regimens were twice the cost of the low-dose regimen.

Disability-adjusted life-years were used as the outcome measure for recurrences and hemolytic events. Disability weights were taken from the Global Burden of Disease study,^29^ and expected remaining years of life for the mean age of adult males and females recruited into the IMPROV trial^5^ was taken from the WHO.^30^ This life expectancy was used to calculate the DALYs due to deaths from *P. vivax* recurrences. Incremental cost-effectiveness ratios were calculated for instances where a treatment strategy cost more money while averting DALYs, where Cost is the total cost of the strategy and DALYs is the total DALYs of the corresponding strategy:

$$\mathrm{ICER}=\;\frac{\mathrm{Cos}t_{1}\mathrm{– Cos}t_{2}}{\mathrm{DAL}Y_{2}\mathrm{-DAL}Y_{1}}.$$

Strategies were ranked according to increasing costs, and interventions that were more expensive while averting fewer DALYs than the previous option (i.e., dominated) were removed. A small ICER corresponding to a smaller investment per DALY averted is more desirable than a large ICER, and the best results are scenarios that avert DALYs while saving costs.

### Sensitivity analyses.

Ranges for all model parameters are presented in addition to the point estimate for the base case. These were taken from the CIs, interquartile ranges, and other values to represent plausible ranges for each value. A one-way sensitivity analysis investigated the impact of this range on the results by varying one parameter at a time. A scenario analysis looked at the societal costs by including the direct household costs of treatment-seeking, treatment, and transport per vivax malaria episode and additional cost of travel per referral to get quantitative testing (Table 1).^26^ An additional scenario analysis examined results where PQ14 was assumed to have efficacy equivalent to PQ7 (i.e., the “increase in recurrences if received PQ14 or TQ (instead of PQ7)” parameter was set to 1). Threshold analyses were also conducted for the following parameters: PQ7 adherence, the quantitative test cost, and the proportion of women who uptake referral to a higher facility for quantitative G6PD testing. In addition, a probabilistic sensitivity analysis (PSA) characterized the uncertainty surrounding the decision to adopt a new strategy by sampling from the full distributions of all parameters (Tables 1 and 2). Results were compared with a willingness-to-pay threshold of one gross domestic product (GDP) per capita per DALY averted.^31^ To reflect recent evidence suggesting that the threshold should be lower,^32^ results were also compared with one-half GDP per capita.

## RESULTS

### Base case results.

All 3 sex-based tafenoquine treatment strategies averted more DALYs than usual care. Across all countries, the treatment of G6PD normal females with high-dose PQ7 had the greatest impact on DALYs and was the only cost-effective option (Table 3). Supplemental Table 1 presents results stratified by sex. In comparison to the PQ7 option, the PQ14 and referral for tafenoquine strategies were dominated (i.e., cost more money and averted fewer DALYs). The ICER for the PQ7 option ranged from $127 in Vietnam to $4,443 in Indonesia when compared with usual care. The higher costs in Indonesia were due to an in-country G6PD RDT unit cost of $13 compared with costs of less than $3 in the other countries. When using a threshold of one GDP per capita, PQ7 was cost-effective in Ethiopia and Vietnam but was only cost-effective for Vietnam when using the lower threshold of one-half GDP per capita.

**Table 3:** Cost-effectiveness results per person from base case and scenario analyses (2016 US$)

Country				Base case analysis					Scenario analyses									
	Strategy			Healthcare provider perspective					Societal perspective					PQ14 and TQ efficacy equal to PQ7				
	G6PD screening	Males	Females	Cost	Incremental costs	DALYs	DALYs averted	ICER	Cost	Incremental costs	DALYs	DALYs averted	ICER	Cost	Incremental costs	DALYs	DALYs averted	ICER
Afghanistan	No	PQ14		$6.1	–	0.0140	–	–	$18.6	–	0.0140	–	–	$5.8	–	0.0131	–	–
	Yes	TQ	PQ7	$11.5	$5.4	0.0091	0.0050	$1,089	$22.6	$4.0	0.0091	0.0050	$815	$10.2	$4.5	0.0077	0.0055	$814
	Yes	TQ	PQ14	$11.7	–	0.0096	–	Dominated	$23.2	–	0.0096	–	Dominated	$10.3	–	0.0080	–	Dominated
	Yes	TQ	Referral for TQ	$12.9	–	0.0095	–	Dominated	$24.8	–	0.0095	–	Dominated	$11.3	–	0.0079	–	Dominated
Ethiopia	No	No radical cure		$11.6	–	0.0181	–	–	$35.5	–	0.0181	–	Dominated	$11.6	–	0.0181	–	–
	Yes	TQ	PQ7	$15.2	$3.6	0.0104	0.0077	$466	$30.9	–	0.0104	–	Cost-saving	$13.7	$2.1	0.0089	0.0092	$228
	Yes	TQ	PQ14	$15.8	–	0.0119	–	Dominated	$33.0	–	0.0119	–	Dominated	$14.0	–	0.0101	–	Dominated
	Yes	TQ	Referral for TQ	$17.9	–	0.0115	–	Dominated	$35.6	–	0.0115	–	Dominated	$15.8	–	0.0096	–	Dominated
Indonesia	No	PQ14		$11.6	–	0.0124	–	–	$83.2	–	0.0124	–	–	$10.9	–	0.0117	–	–
	Yes	TQ	PQ7	$30.2	$18.6	0.0082	0.0042	$4,443	$95.0	$11.9	0.0082	0.0042	$2,828	$28.0	$17.1	0.0073	0.0044	$3,925
	Yes	TQ	PQ14	$31.2	–	0.0089	–	Dominated	$98.6	–	0.0089	–	Dominated	$28.4	–	0.0079	–	Dominated
	Yes	TQ	Referral for TQ	$34.4	–	0.0087	–	Dominated	$102.3	–	0.0087	–	Dominated	$31.4	–	0.0076	–	Dominated
Vietnam	No	PQ14		$11.7	–	0.0160	–	–	$52.3	–	0.0160	–	Dominated	$11.2	–	0.0152	–	Dominated
	Yes	TQ	PQ7	$12.6	$0.9	0.0092	0.0068	$127	$44.4	–	0.0092	–	Cost-saving	$11.2	–	0.0077	–	Cost-saving
	Yes	TQ	PQ14	$12.8	–	0.0096	–	Dominated	$45.1	–	0.0096	–	Dominated	$11.2	–	0.0080	–	Dominated
	Yes	TQ	Referral for TQ	$13.1	–	0.0095	–	Dominated	$45.8	–	0.0095	–	Dominated	$11.6	–	0.0079	–	Dominated

DALYs = disability-adjusted life-years; ICER = incremental cost-effectiveness ratio; G6PD = glucose-6-phosphate dehydrogenase; PQ7 = 7-day high-dose primaquine; PQ14 = 14-day low-dose primaquine; TQ = tafenoquine. The first strategy listed for each country is current usual care. See Supplemental Table 1 for results by sex.

### One-way sensitivity analysis results.

**Figure 2.**
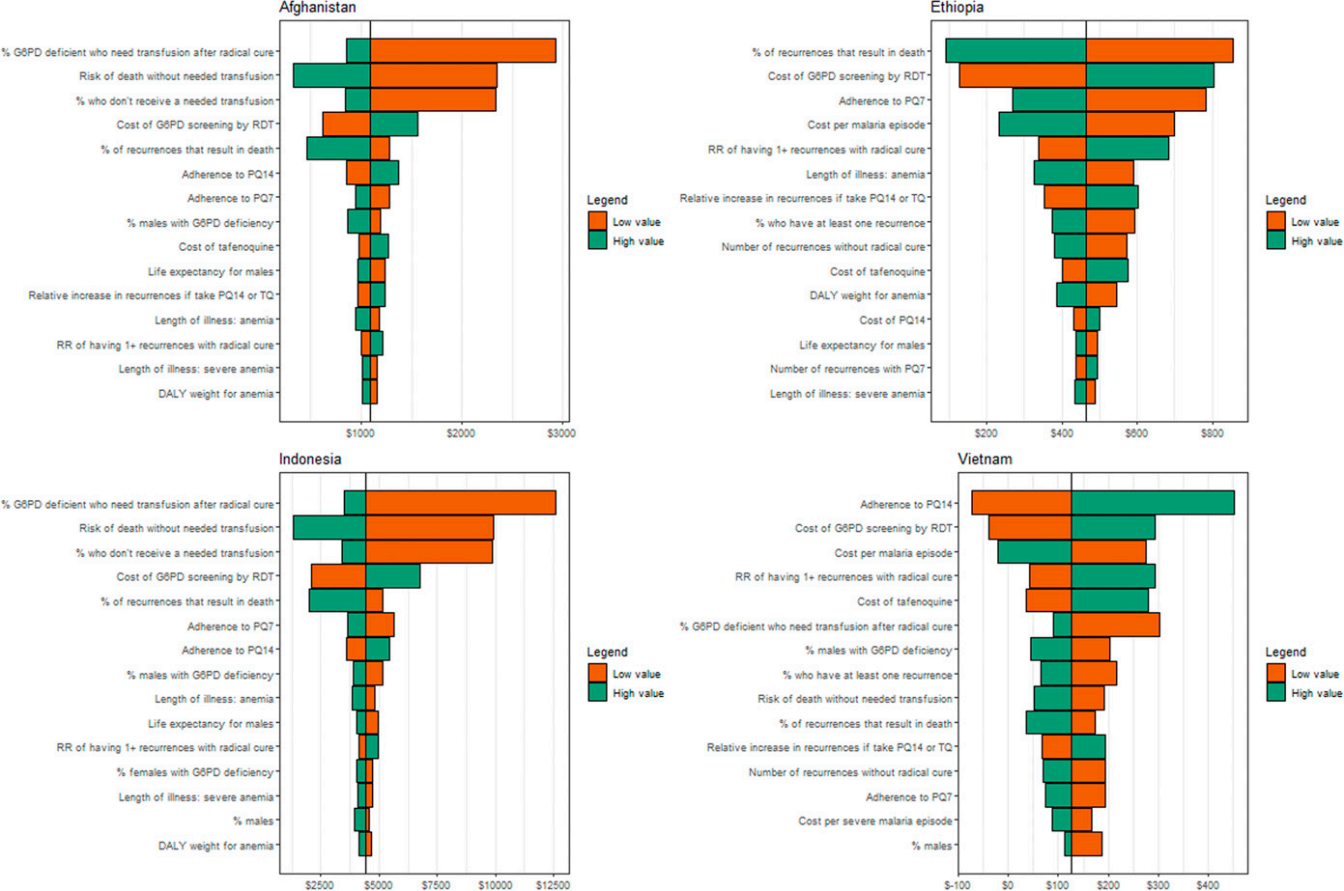
Tornado diagrams of the one-way sensitivity analysis for the comparison of the treatment strategies using PQ7 to usual care. The black vertical line corresponds to the baseline ICER in each country, and the colored bars correspond to resulting ICER when each parameter is set to their lower or upper value (base and range values provided in Tables 1 and 2). DALYs = disability-adjusted life-years; G6PD = glucose-6-phosphate dehydrogenase; PQ7 = 7-day high-dose primaquine; PQ14 = 14-day low-dose primaquine; RDT = rapid diagnostic test; TQ = tafenoquine. This figure appears in color at www.ajtmh.org.

The one-way sensitivity analysis showed similar results across all countries for the comparison between usual care and the sex-based treatment strategy with PQ7 (Figure 2). In Afghanistan and Indonesia, the parameters with the largest impact on the results were those relating to hemolysis, the cost of the G6PD RDT, *P. vivax* mortality, and adherence to primaquine. Lowering the cost of the G6PD RDT resulted in PQ7 becoming cost-effective in Indonesia. In Ethiopia, *P. vivax* mortality, G6PD RDT and *P. vivax* episode costs, and adherence to PQ7 had the greatest impact. Adherence to PQ14, the relative risk of recurrence with radical cure, and the costs of G6PD RDTs, *P. vivax* episodes, and tafenoquine resulted in the greatest change in results in Vietnam.

### Scenario and threshold analyses results.

From a societal perspective, in which costs incurred by affected households were also included, the incremental costs of all three sex-based treatment strategies were lower across all countries (Table 3). In Ethiopia and Vietnam, all strategies saved costs compared with the current practice. Because the PQ7 option averted the most DALYs in both of those countries, it dominated usual care. The PQ7 option remained the only cost-effective option in Afghanistan and Indonesia, with lowered ICERs of $815 and $2,828, respectively.

In the scenario analysis where TQ and PQ14 were assumed to have equal efficacy to PQ7, costs and DALYs were reduced for all strategies (Table 3). Because of the increased efficacy of tafenoquine in males, the DALYs averted by the PQ7 option increased in comparison with usual care, whereas the incremental costs decreased for all countries. Consequently, the ICERs decreased to $814 in Afghanistan, $228 in Ethiopia, and $3,925 in Indonesia. In Vietnam, the decrease in PQ7 costs resulted in overall cost savings, so it dominated usual care. When setting the referral uptake to 100%, the strategy of referral had similar DALYs as the PQ7 option; however, the costs remained higher across all countries (results not shown). The referral option was dominated even when setting the cost of quantitative G6PD screening to $0. The threshold analysis found that the PQ7 intervention became cost-effective at a willingness-to-pay threshold of 1 GDP per capita when adherence was increased to 97% in Indonesia. It was not cost-effective for Afghanistan or at a threshold of one-half GDP per capita.

### Probabilistic sensitivity analysis results.

**Figure 3.**
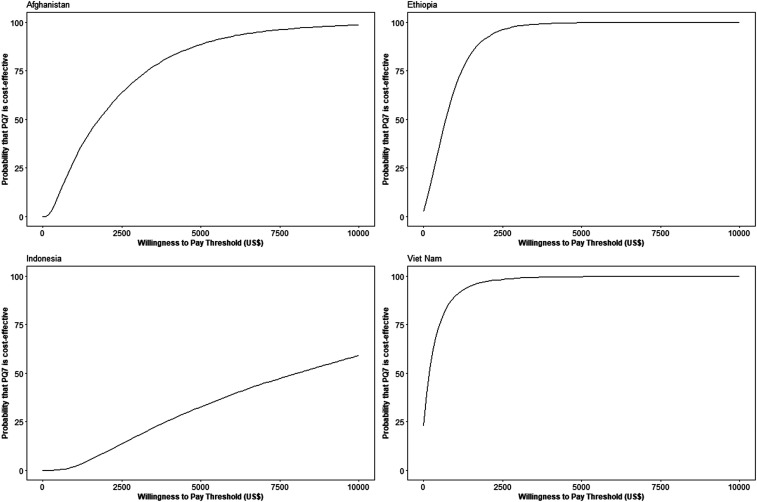
Cost-effectiveness acceptability curves for the comparison of the sex-based treatment strategy with tafenoquine for males and 7-day high-dose primaquine (PQ7) for females to usual care.

Figure 3 presents the decision uncertainty in cost-effectiveness acceptability curves. These curves show the strength of evidence that the sex-based strategy with PQ7 intervention is cost-effective for willingness-to-pay thresholds up to $10,000 per DALY averted. For Afghanistan, Ethiopia, and Vietnam, the PQ7 strategy showed a high probability of cost-effectiveness for reasonably low willingness-to-pay thresholds. For Indonesia, the probability that it would be cost-effective at a threshold of $10,000 was only 59%. When using a threshold of one GDP per capita,^31^ the PQ7 strategy had a 15% probability of being cost-effective in Afghanistan ($600), 50% in Ethiopia ($700), 23% in Indonesia ($3,600), and 98% in Vietnam ($2,200). The scatterplots from the PSA are provided in Supplemental Figure 1.

## DISCUSSION

In most countries, the main burden of disease caused by *P. vivax* occurs in rural areas with poor and remote healthcare systems where it is difficult to access care. Accordingly, it is likely that the stringent safety regulations for tafenoquine will mean that few patients with vivax malaria will be able to receive it.^21^ If deemed safe and appropriate, sex-based treatment strategies have potential to be cost-effective, particularly in settings where the G6PD RDT is accessible at a low cost. These sex-based strategies would also be useful for areas where a quantitative G6PD test is not yet available. As shown by the results from Vietnam, where G6PD prevalence in males was estimated to be 5%, the impact would likely be greatest in areas such as the Greater Mekong Subregion, where most patients with malaria are males who can be reliably diagnosed with the G6PD RDT and thus be treated safely with tafenoquine.

The different scenarios demonstrate roughly similar reductions in DALYs (approximately a third of current strategies for usual care) and similar costs associated with all three strategies, even assuming only a 50% uptake rate by women for quantitative testing. Although the option where females are treated with PQ7 emerged as the optimal strategy in terms of cost-effectiveness, changing the assumptions on adherence, efficacy, and uptake of referrals to align with local situations had major effects on results, indicating that in some scenarios, the PQ14 or referral option could be more cost-effective. Efforts to bring TQ for males who test normal by G6PD RDT into policy should be explored as a strategy to fast-track progress toward current elimination goals.

Several studies have shown that a high proportion of recurrent *P. vivax* episodes are attributable to relapse.^17,33,34^ One mathematical model estimated that treating only 60% of cases with effective radical cure would lead to elimination in 10 years even in a low relapse setting such as northwest India.^17^ This indicates that even partial coverage with radical cure could have a significant impact on *P. vivax* transmission. Decreased transmission also provides an opportunity to protect vulnerable populations, including pregnant women and children younger than 6 months, for whom radical cure is contraindicated. Anemia and malnutrition associated with these relapses will also be reduced, which will be of particular benefit to children. These benefits are not explicitly captured in this model but would increase the cost-effectiveness of the sex-based treatment strategies.

Our analysis has several limitations. First is the assumption that the efficacy of high-dose PQ7 is significantly greater than that of both tafenoquine and low-dose PQ14. This assumption is likely to vary markedly by location, although few trials have compared low- and high-dose primaquine regimens directly.^35^ Primaquine effectiveness is impacted not only by the dosing but also by the adherence of patients to the regimen for which robust data are also limited.^23–25,36^ We assumed that in the referral strategy, women not using their referrals would not be given the option of primaquine at their initial consultation. Although this was chosen because it was economically conservative and programmatically straightforward, it would be problematic because it involves denying women the standard of care in Afghanistan, Indonesia, and Vietnam. If primaquine were prescribed to women who do not want the referral, it would improve the outcomes of the referral strategy. In most of the countries included in our analysis, primaquine is recommended without G6PD testing; however, it is often not used because of the fears of drug-induced hemolysis. Our model did not account for the risk of hemolysis in heterozygous women, although a clinical trial has shown that this can occur.^13^

The G6PD deficiency frequencies were taken from a whole-population model, instead of from patients presenting with vivax malaria. Because G6PD deficiency can have a protective effect against malaria, the prevalence of G6PD deficiency may have been overestimated.^37^ Strengthening the evidence for the base case parameter values and narrowing the ranges would increase the certainty of the cost-effectiveness estimates. Although the CareStart RDT has shown good performance in research studies,^16^ there have been difficulties reported in field settings due to the lack of a control indicator, and users have struggled to interpret the faint-positive results.^38^ Accordingly, healthcare workers using these tests will require thorough training until a more user-friendly RDT becomes available. Finally, the G6PD RDT has temperature limitations similar to malaria RDTs.

Outside sub-Saharan Africa, the safe and effective radical cure of *P. vivax* has potential to have a major impact on the burden of malaria. For the foreseeable future, treatment options all come with a risk of hemolysis that must be dealt with in all sectors of the health system. The current need is to optimize the use of available tools. Even if tafenoquine can only be safely given to a fraction of the population, it would provide some protective effect on those unable to receive radical cure, and its impact on transmission could be substantial enough to pave way for elimination.^39^ Using a treatment approach that centers on G6PD testing via RDT followed by sex specific treatment strategies, while requiring a robust supply chain for several products, has potential to ensure the widespread use of tafenoquine without major infrastructure adjustments. Importantly, this approach is not an alternative to the general widespread rollout of tafenoquine and quantitative G6PD testing but is a proposal on how lower resource settings can bridge between usual care and a full rollout of quantitative G6PD screening with tafenoquine. Using a sex-based treatment strategy as an interim solution could significantly change the landscape for providing the radical cure of *P. vivax*, an essential step for the ultimate elimination of malaria.

## Supplemental appendix, table and figure

Supplemental materials

## References

[1] Battle KE 2019 Mapping the global endemicity and clinical burden of *Plasmodium vivax*, 2000–17: a spatial and temporal modelling study. Lancet 394: 332–343. 31229233 10.1016/S0140-6736(19)31096-7 PMC6675736

[2] Baird JK Hoffman SL, 2004 Primaquine therapy for malaria. Clin Infect Dis 39: 1336–1345. 15494911 10.1086/424663

[3] Thriemer K 2018 Quantifying primaquine effectiveness and improving adherence: a round table discussion of the APMEN Vivax Working Group. Malar J 17: 241. 29925430 10.1186/s12936-018-2380-8 PMC6011582

[4] Douglas NM Poespoprodjo JR Patriani D Malloy MJ Kenangalem E Sugiarto P Simpson JA Soenarto Y Anstey NM Price RN, 2017 Unsupervised primaquine for the treatment of *Plasmodium vivax* malaria relapses in southern Papua: a hospital-based cohort study. PLoS Med 14: e1002379. 28850568 10.1371/journal.pmed.1002379 PMC5574534

[5] Taylor WRJ 2019 Short-course primaquine for the radical cure of *Plasmodium vivax* malaria: a multicentre, randomised, placebo-controlled non-inferiority trial. Lancet 394: 929–938. 31327563 10.1016/S0140-6736(19)31285-1 PMC6753019

[6] Chu CS 2018 Comparison of the cumulative efficacy and safety of chloroquine, artesunate, and chloroquine-primaquine in *Plasmodium vivax* malaria. Clin Infect Dis 67: 1543–1549. 29889239 10.1093/cid/ciy319 PMC6206118

[7] Lacerda MVG 2019 Single-dose tafenoquine to prevent relapse of *Plasmodium vivax* malaria. N Engl J Med 380: 215–228. 30650322 10.1056/NEJMoa1710775 PMC6657226

[8] Llanos-Cuentas A 2019 Tafenoquine versus primaquine to prevent relapse of *Plasmodium vivax* malaria. N Engl J Med 380: 229–241. 30650326 10.1056/NEJMoa1802537 PMC6657225

[9] Howes RE 2012 G6PD deficiency prevalence and estimates of affected populations in malaria endemic countries: a geostatistical model-based map. PLoS Med 9: e1001339. 23152723 10.1371/journal.pmed.1001339 PMC3496665

[10] World Health Organization, 2016 Testing for G6PD Deficiency for Safe Use of Primaquine in Radical Cure of *P. Vivax* and *P. Ovale* Malaria. Geneva, Switzerland: WHO.

[11] Domingo GJ 2013 G6PD testing in support of treatment and elimination of malaria: recommendations for evaluation of G6PD tests. Malar J 12: 391. 24188096 10.1186/1475-2875-12-391 PMC3830439

[12] Ley B 2015 The challenges of introducing routine G6PD testing into radical cure: a workshop report. Malar J 14: 377. 26416229 10.1186/s12936-015-0896-8 PMC4587750

[13] Chu CS Bancone G Nosten F White NJ Luzzatto L, 2018 Primaquine-induced haemolysis in females heterozygous for G6PD deficiency. Malar J 17: 101. 29499733 10.1186/s12936-018-2248-y PMC5833093

[14] World Health Organization, 2015 Guidelines for the Treatment of Malaria. Geneva, Switzerland: WHO.

[15] Ley B Bancone G von Seidlein L Thriemer K Richards JS Domingo GJ Price RN, 2017 Methods for the field evaluation of quantitative G6PD diagnostics: a review. Malar J 16: 361. 28893237 10.1186/s12936-017-2017-3 PMC5594530

[16] Ley B 2019 Performance of the Access Bio/CareStart rapid diagnostic test for the detection of glucose-6-phosphate dehydrogenase deficiency: a systematic review and meta-analysis. PLoS Med 16: e1002992. 31834890 10.1371/journal.pmed.1002992 PMC6910667

[17] Roy M Bouma MJ Ionides EL Dhiman RC Pascual M, 2013 The potential elimination of *Plasmodium vivax* malaria by relapse treatment: insights from a transmission model and surveillance data from NW India. PLoS Negl Trop Dis 7: e1979. 23326611 10.1371/journal.pntd.0001979 PMC3542148

[18] Devine A Parmiter M Chu CS Bancone G Nosten F Price RN Lubell Y Yeung S, 2017 Using G6PD tests to enable the safe treatment of *Plasmodium vivax* infections with primaquine on the Thailand-Myanmar border: a cost-effectiveness analysis. PLoS Negl Trop Dis 11: e0005602. 28542194 10.1371/journal.pntd.0005602 PMC5460895

[19] Gilder ME 2018 Primaquine pharmacokinetics in lactating women and breastfed infant exposures. Clin Infectious Dis 67: 1000–1007. 10.1093/cid/ciy235 PMC6137118 29590311

[20] Pal S 2019 Evaluation of a novel quantitative test for glucose-6-phosphate dehydrogenase deficiency: bringing quantitative testing for glucose-6-phosphate dehydrogenase deficiency closer to the patient. Am J Trop Med Hyg 100: 213–221. 30350771 10.4269/ajtmh.18-0612 PMC6335905

[21] White NJ, 2019 Tafenoquine–a radical improvement? N Engl J Med 380: 285–286. 30650321 10.1056/NEJMe1816383

[22] Abreha T 2017 Comparison of artemether-lumefantrine and chloroquine with and without primaquine for the treatment of *Plasmodium vivax* infection in Ethiopia: a randomized controlled trial. PLoS Med 14: e1002299. 28510573 10.1371/journal.pmed.1002299 PMC5433686

[23] Grietens KP 2010 Adherence to 7-day primaquine treatment for the radical cure of *P. vivax* in the Peruvian Amazon. Am J Trop Med Hyg 82: 1017–1023. 20519594 10.4269/ajtmh.2010.09-0521 PMC2877405

[24] Cheoymang A Ruenweerayut R Muhamad P Rungsihirunrat K Na-Bangchang K, 2015 Patients’ adherence and clinical effectiveness of a 14-day course of primaquine when given with a 3-day chloroquine in patients with *Plasmodium vivax* at the Thai-Myanmar border. Acta Trop 152: 151–156. 26278026 10.1016/j.actatropica.2015.08.008

[25] Khantikul N Butraporn P Kim HS Leemingsawat S Tempongko MA Suwonkerd W, 2009 Adherence to antimalarial drug therapy among vivax malaria patients in northern Thailand. J Health Popul Nutr 27: 4–13. 19248643 10.3329/jhpn.v27i1.3313 PMC2761802

[26] Devine A 2019 Provider and household costs of *Plasmodium vivax* malaria episodes: a multicountry comparative analysis of primary trial data. Bull World Health Organ 97: 828–836. 31819291 10.2471/BLT.18.226688 PMC6883272

[27] World Health Organization, 2011 WHO-CHOICE Unit Cost Estimates for Service Delivery. Available at: http://www.who.int/choice/cost-effectiveness/inputs/health_service/en/. Accessed February 2, 2016.

[28] Mulligan J-A Fox-Rushby JA Adam T Johns B Mills A, 2005 Unit Costs of Health Care Inputs in Low and Middle Income Regions. DCPP Working Paper No. 9.

[29] Salomon JA 2013 Common values in assessing health outcomes from disease and injury: disability weights measurement study for the Global Burden of Disease Study 2010. Lancet 380: 2129–2143. 10.1016/S0140-6736(12)61680-8 PMC10782811 23245605

[30] World Health Organization, 2016 Life Tables by Country. Available at: http://apps.who.int/gho/data/node.main.LIFECOUNTRY?lang=en. Accessed November 11, 2019.

[31] The World Bank, 2016 GDP per Capita (Current US$) Available at: http://data.worldbank.org/indicator/NY.GDP.PCAP.CD. Accessed August 27, 2019.

[32] Ochalek J Lomas J Claxton K, 2018 Estimating health opportunity costs in low-income and middle-income countries: a novel approach and evidence from cross-country data. BMJ Global Health 3: e000964. 10.1136/bmjgh-2018-000964 PMC6231096 30483412

[33] Taylor AR 2019 Resolving the cause of recurrent *Plasmodium vivax* malaria probabilistically. Nat Commun 10: 5595. 31811128 10.1038/s41467-019-13412-x PMC6898227

[34] Adekunle AI Pinkevych M McGready R Luxemburger C White LJ Nosten F Cromer D Davenport MP, 2015 Modeling the dynamics of *Plasmodium vivax* infection and hypnozoite reactivation in vivo. PLoS Negl Trop Dis 9: e0003595. 25780913 10.1371/journal.pntd.0003595 PMC4364305

[35] John GK Douglas NM von Seidlein L Nosten F Baird JK White NJ Price RN, 2012 Primaquine radical cure of Plasmodium vivax: a critical review of the literature. Malar J 11: 280. 22900786 10.1186/1475-2875-11-280 PMC3489597

[36] Bruxvoort K Goodman C Kachur SP Schellenberg D, 2014 How patients take malaria treatment: a systematic review of the literature on adherence to antimalarial drugs. PLoS One 9: e84555. 24465418 10.1371/journal.pone.0084555 PMC3896377

[37] Mbanefo EC 2017 Association of glucose-6-phosphate dehydrogenase deficiency and malaria: a systematic review and meta-analysis. Sci Rep 7: 45963. 28382932 10.1038/srep45963 PMC5382680

[38] Henriques G 2018 Comparison of glucose-6 phosphate dehydrogenase status by fluorescent spot test and rapid diagnostic test in Lao PDR and Cambodia. Malar J 17: 243. 29929514 10.1186/s12936-018-2390-6 PMC6013858

[39] Brummaier T Gilder ME Gornsawun G Chu CS Bancone G Pimanpanarak M Chotivanich K Nosten F McGready R, 2020 Vivax malaria in pregnancy and lactation: a long way to health equity. Malar J 19: 40. 10.1186/s12936-020-3123-1 PMC6977346 31969155

[40] Pamba A Richardson ND Carter N Duparc S Premji Z Tiono AB Luzzatto L, 2012 Clinical spectrum and severity of hemolytic anemia in glucose 6-phosphate dehydrogenase-deficient children receiving dapsone. Blood 120: 4123–4133. 22993389 10.1182/blood-2012-03-416032

[41] Rahimi BA Thakkinstian A White NJ Sirivichayakul C Dondorp AM Chokejindachai W, 2014 Severe vivax malaria: a systematic review and meta-analysis of clinical studies since 1900. Malar J 13: 481. 25486908 10.1186/1475-2875-13-481 PMC4364574

