# Supplementary figures and images for: Cost-Effectiveness Analysis of Sex-Stratified Plasmodium vivax Treatment Strategies Using Available G6PD Diagnostics to Accelerate Access to Radical Cure
Source: Am J Trop Med Hyg. 2020 May 4;103(1):394–403. doi: 10.4269/ajtmh.19-0943 (PMC7356471; doi:10.4269/ajtmh.19-0943)

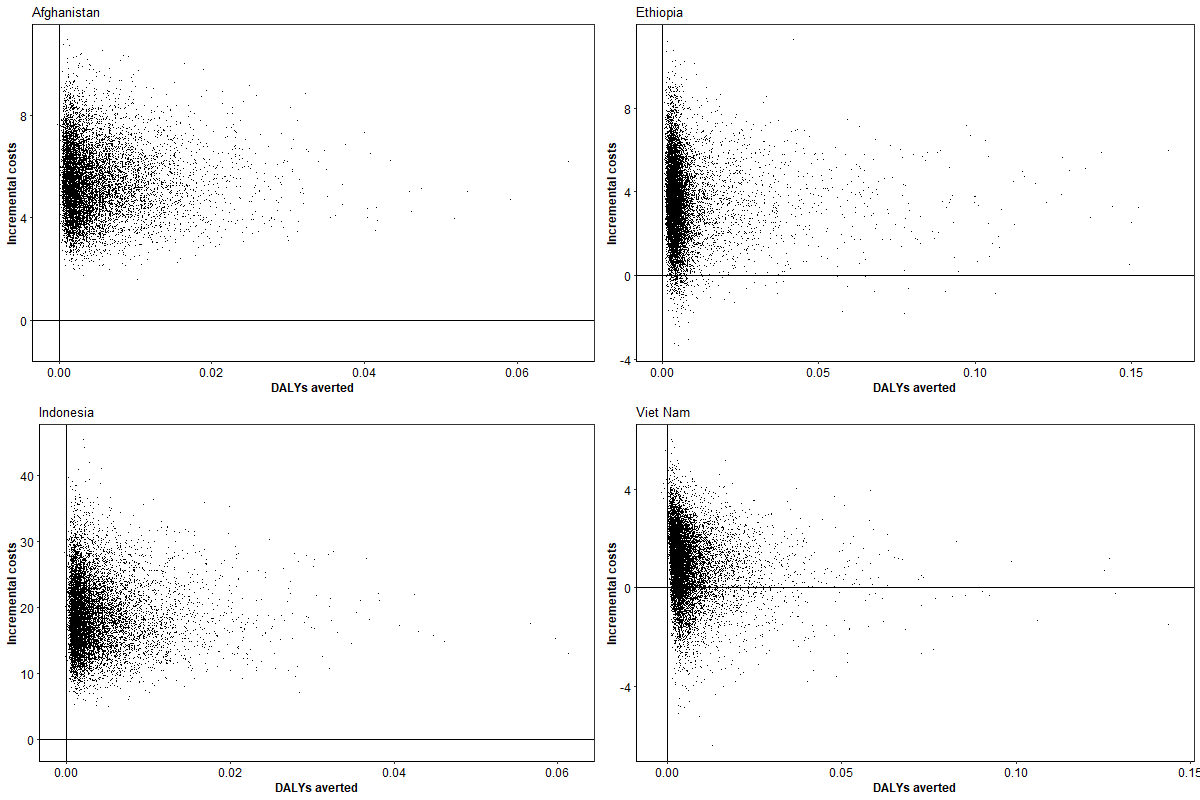

Supplement: Supplementary file 1 [file tpmd190943.SD1.png]
