# Supplementary material for: Cost-Effectiveness Analysis of Sex-Stratified Plasmodium vivax Treatment Strategies Using Available G6PD Diagnostics to Accelerate Access to Radical Cure
Source: Am J Trop Med Hyg. 2020 May 4;103(1):394–403. doi: 10.4269/ajtmh.19-0943 (PMC7356471; doi:10.4269/ajtmh.19-0943)
Supplement: Supplementary file 2 [file tpmd190943.SD2.docx]

**SUPPLEMENTAL APPENDIX**

**Equations for vivax malaria recurrences over a one-year time horizon adjusting for adherence rates.**

| **Symbol** | **Definition** |
| --- | --- |
| $\varphi$ | Proportion with at least one relapse |
| *RR* | Relative risk of relapse if receive radical cure |
| $N_{b}$ | Mean number of relapses if only receive blood stage treatment |
| $N_{r}$ | Mean number of relapses if receive radical cure |
| $\alpha_{h}$ | Proportion adhere to 7-day high-dose primaquine |
| $\alpha_{l}$ | Proportion adhere to 14-day low-dose primaquine |
| $\alpha_{w}$ | Proportion adhere to 8-weekly primaquine |
| $\mu$ | Relative increase in relapses with low-dose versus high-dose primaquine |

Recurrences in those not receiving radical cure:

$\varphi\times N_{b}$

Recurrences in those prescribed 7-day high-dose primaquine:

$$(\varphi\times RR\times N_{r}\times\alpha_{h})+(\varphi\times N_{b}\times\left( 1-\alpha_{h} \right))$$

Recurrences in those prescribed 14-day low-dose primaquine:

$$(\varphi\times RR\times N_{r}\times\alpha_{l}\times\mu)+(\varphi\times N_{b}\times\left( 1-\alpha_{l} \right))$$

Recurrences in those prescribed 8 weekly doses of primaquine:

$$(\varphi\times RR\times N_{r}\times\alpha_{w}\times\mu)+(\varphi\times N_{b}\times\left( 1-\alpha_{w} \right))$$

Recurrences in those prescribed tafenoquine:

$$\varphi\times RR\times N_{r}\times\mu$$

**Supplemental Figure 1.** Scatter plots from probabilistic sensitivity analysis for the comparison of the sex-based treatment algorithm with 7-day high-dose primaquine for females and tafenoquine for males to current practice (no G6PD screening and 14-day low-dose primaquine for all countries except Ethiopia where PQ is not given). All costs are in 2016 United States Dollars.

**Supplemental Table 1.**
